# A Remarkable Instance of a Patient's Recovery after Taking a Very Large Dose of Corrosive Sublimate

**Published:** 1785

**Authors:** 


V. *A remarkable Inſtance of a Patient's Recovery after taking a very large Doſe of corroſive Sublimate. Communicated in a Letter to Dr.* Simmons, *by* Thomas Houlſton, *M. D. Phyſician to the Liverpool Infirmary.*

I AM induced to ſend you a conciſe account of a caſe which fell lately under my notice, as it affords, in my opinion, a very ſtriking proof of the advantages to be expected from alkaline medicines ſpeedily adminiſtered, and ſteadily perſevered in.

A ſhip ſurgeon here, on the 9th of April laſt, at one in the morning, mixed ſix drachms of corroſive ſublimate in a tumbler glaſs of water, drank it, and, as ſome remained at the bottom of the glaſs, he rinſed it twice with water, which he alſo ſwallowed. Something leſs leſs than two drachms of the ſublimate remained at the bottom of the glaſs; but he certainly took about half an ounce.

He repented almoſt immediately of what he had done, drank plentifully of warm water, and, three quarters of an hour afterwards, took three tea-cupfuls of oil. He ſoon vomited; and proper aſſiſtance was expeditiouſly procured.

A ſolution of ſalt of tartar was very judiciouſly exhibited, and continued during the day, when it was thought eligible to remove him to the Infirmary.

His ſufferings were great, and aggravated by his anxious deſire of life; his diſcharges upwards and downwards very frequent, and mixed with a good deal of blood.

In conſultation, at the Infirmary, the plan fixed upon and purſued was, a continuance of the ſolution of an alkali occaſionally by the mouth, and the ſame thrown up forcibly in a large clyſter, and, in like manner, the tinctura thebaica; neither of which, however, were long retained*. For the latter, the opium in ſubſtance

* Beſides theſe, the warm bath was uſed three times; fomentations were applied to the ſtomach; and, before his admiſſion fubftance was fubftituted; and for the alkaline falt, (the next day) the calcined magnefia*: for he manifefted a repugnance to the folution of falt of tartar, (as he has fince told me) folely on account of its naufeous tafte; and it alfo feemed to caufe fome irritation of the ftomach, which was foon followed by vomiting: difagreeable fenfations, no doubt, but of little confequence, and fcarcely meriting attention, where the object is of fuch magnitude as fnatching a victim from the jaws of death. Nor is it at all probable but that the fame, and even greater degrees of pain and irritation muft have arifen from the mere action of the fublimate on the ftomach, *in proportion as it diffolved*, which action would be prevented and counteracted by the alkaline folution, was it retained only for a moment.

On a farther folution of the fublimate, a frefh exhibition of the alkali becomes necefmiffion into the infirmary, a fingle dofe of ipecac. (℈ij.) had been given.

* He took near five ounces of falt of tartar in about thirty hours: it was then changed for the magnefia, and afterwards his vomitings were lefs frequent, but recurred occafionally for five days. It is obfervable that no degree of ptyalifm ever took place.

 fary;

ſary; and there does not ſeem to be any well-grounded objection to its uſe under ſo ſore and tender a ſtate of the ſtomach, ſince, contrary to all reaſonable expectation, in leſs than a week he got perfectly well, and, notwithſtanding the hæmorrhage, did not feel any degree of uneaſineſs in the ſtomach or bowels. He left the houſe ſoon afterwards, thoroughly ſenſible of the benefit he had received, and expreſſing himſelf penitent for the evil he had attempted.

*Liverpool,*

May 1, 1785.

